# Exclusive Fish Oil Lipid Emulsion Rescue Strategy Improves Cholestasis in Neonates on Partially Fish Oil-Based Lipid Emulsion: A Pilot Study

**DOI:** 10.3390/nu15030509

**Published:** 2023-01-18

**Authors:** David Ramiro-Cortijo, Sonia Del Pozo Arribas, Lidia Inisterra Viu, Natalia García Vázquez, Miguel Saenz de Pipaon

**Affiliations:** 1 Department of Physiology, Faculty of Medicine, C/Arzobispo Morcillo 2, Universidad Autónoma de Madrid, 28029 Madrid, Spain; 2 Department of Neonatology, Instituto de Investigación Sanitaria Hospital Universitario La Paz (IdiPAZ), Hospital Universitario La Paz, Paseo de la Castellana 261, 28046 Madrid, Spain; 3 Department of Pediatrics, Hospital Universitario Puerta de Hierro, C/Joaquín Rodrigo 1, Majadahonda, 28222 Madrid, Spain; 4 Department of Pharmacy, Hospital Universitario La Paz, Paseo de la Castellana 261, 28046 Madrid, Spain

**Keywords:** parenteral nutrition, liver disease, omega-3 fatty acids, preterm infant, fat emulsions

## Abstract

Resolution of parenteral nutrition-associated liver disease has been identified in infants receiving SMOFlipid™ or a 100% fish oil lipid emulsion (FOLE). However, the effect of FOLE is unknown when the previous emulsion received is a mixed lipid emulsion containing fish oil. This observational pilot study reports data regarding the use of Omegaven™ after the diagnosis of cholestasis while receiving SMOFlipid™. We conducted a retrospective review of medical charts of neonates in which a partially fish oil-based lipid emulsion was replaced by a fish oil lipid emulsion at 1 g/kg/day due to cholestasis. Thirty-eight infants (92.1% preterm, being 44.7% born below 28 weeks’ gestation), received FOLE. Birth weight was 1390 (743.0; 2298) grams. The age that cholestasis diagnosed was 15.0 (10.0; 24.8) days. The fish oil emulsion was administered for 38.5 (11.2; 51.8) days. In 73.7% (28/38) of the neonates, the cholestasis was resolved. In 34.2% (13/38), resolution happened before FOLE discontinuation. In addition, in the rest of the neonates (15) in whom cholestasis resolved, resolution occurred after FOLE discontinuation. Nine of the neonates died. In conclusion, the use of a 100% fish oil-based emulsion in neonates afflicted with cholestasis developed while on a partially fish oil-based emulsion is associated with a bilirubin decrease.

## 1. Introduction

Parenteral nutrition (PN) is a therapy that intravenously delivers fluids and essential nutrients (electrolytes, carbohydrates, amino acids, and micronutrients) to patients who are unable to meet their nutrition requirements via standard enteral feeding. In infants, PN is a key part of the treatment in the neonatal intensive care units (NICU), being critical to the survival of preterm infants as well as infants with underlying gastrointestinal pathology that prevents full enteral feeding. PN was first established as a life-saving approach for nutritional support in infants in 1968. Intravenous lipid emulsions complement PN, being a source of non-protein calories and fatty acids. PN should provide an appropriate amount of carbohydrates, protein, and fat to ensure optimal growth and development. Line infections and sepsis are the most common issues in PN. However, prolonged use of PN can be associated with adverse clinical outcomes, most notably liver injury in the form of PN-associated liver disease (PNALD) that manifests initially as cholestasis, defined as an elevated serum conjugated bilirubin >2 mg/dL [1,2]. In neonates, the incidence of PNALD is up to 85% when prolonged PN is required [3]. The infants whom PNALD is poorly treated may lead to liver cirrhosis, irreversible liver failure and require liver transplantation associated with high morbidity and mortality. Furthermore, there are limited strategies to manage PNALD. The most effective measure would be to establish exclusive enteral nutrition and discontinue the PN. However, this is extremely difficult to achieve in certain situations [4]. 

The PNALD in pediatric patients is usually multifactorial due to nutritional and non-nutritional causes, sepsis (through bacterial translocation or central line infections), infant prematurity, and immature liver function. The role of lipid therapy, as a contributing cause, is well-established with the pathophysiological pathways now well understood. Lipid emulsion composition is a modifiable factor related to PNALD that can improve disease resolution. Traditional lipid emulsions are composed of soybean oil. Several studies indicate an association between the use of soybean-fat emulsions with PNALD, due to its proinflammatory characteristics related to the impairment of the bile flow [5,6]. Soybeans have an exceptionally high content of linoleic acid (50% of fatty acids), an omega-6 fatty acid. Phytosterol toxicity is the main pathogenic mechanism for lipid-induced liver injury. Phytosterols, sitosterol, stigmasterol, and campesterol, unlike cholesterol, a similarly structured sterol, are not endogenously synthesized. Soybean oil is also rich in phytosterols, plant-derived compounds that reduce biliary flow and contribute to PNALD [6]. To limit toxicities associated with soybean oil, new generation lipid emulsions with soy oil have been partially substituted by olive oil or a combination of medium chain triglycerides (MCT), olive, and fish oils. These new generation lipid emulsions contain a greater amount of omega-3 fatty acids and lower concentration of omega-6 fatty acids and phytosterols [6]. While the omega-6 fatty eicosanoids are more likely to be proinflammatory, the omega-3 eicosanoids could reduce inflammation. High omega-6 to omega-3 ratios, combined with a lack of antioxidant protection, excite inflammation and oxidative stress.

A 100%-fish oil-based lipid emulsion is also available. Resolution of cholestasis and improvement of inflammatory liver parameters in patients with PNALD after a soybean-based lipid emulsion was substituted by a 100% based fish oil lipid emulsion (FOLE) without clinical adverse effects has been reported [7]. There were initial concerns with the use of low-dose FOLE on the development of essential fatty acid deficiency given the very high eicosapentanoic acid and docosahexaenoic acid and very low linoleic acid and alpha-linoleic acid fatty acid composition. However, given at the appropriate daily dose (1 g/kg), FOLE is effective at maintaining essential fatty acid concentrations [6]. In 2018, the Food and Drug Administration approved fish oil monotherapy for the management of pediatric PNALD. However, there are scarce data in the literature regarding the rescue use of a FOLE in PNALD neonates who were already on a partially fish oil-based lipid emulsion (p-FOLE). In this article, we present the clinical experience in a NICU in which FOLE was used as a rescue nutritional strategy in patients who developed PNALD while receiving p-FOLE. Most infants develop PNALD following intestinal failure, which requires prolonged full-PN administration. Not only infants with NEC, meconium ileus, or other intestinal disease were included; we also included infants that develop cholestasis without gut disease while receiving PN. These infants were also included in previous reports and PN was needed [7]. Clinical and nutritional characteristics were compared between infants in which cholestasis resolved after treatment with FOLE and those that persisted.

## 2. Materials and Methods

### 2.1. Study Design and Clinical Outcomes

This was a retrospective pilot study that reviewed the medical records of neonates admitted into a NICU of Hospital Universitario La Paz (HULP, Madrid, Spain), which is considered a Spanish hospital of the third level. Reviewed neonates were admitted in the NICU between January 2014 to August 2018. This study was conducted according to the principles of the Declaration of Helsinki, with the approval of the Ethical Committee of Hospital Universitario La Paz (Ref. PI-1969).

The total simple size was 38 neonates. In addition, these newborns received a FOLE (Omegaven™, Fresenius Kabi AB, Bad Homburg, Germany) after being diagnosed with cholestasis. Additionally, a partially fish oil-based lipid emulsion (15% fish oil; SMOFlipid™, Fresenius Kabi AB, Bad Homburg, Germany) was the first line of the lipid emulsion used during the study period. As a NICU protocol of HULP, SMOFlipid™ was initially started at 1 g/kg/day and progressed daily up to 3 g/kg/day as maximum. In this NICU, Omegaven™ was offered as a compassionate treatment in those neonates who develop cholestasis (defined as conjugated bilirubin levels >4 mg/dL (68.4 µmol/L) in neonates without intestinal disease, or >2 mg/dL (34.2 µmol/L) in neonates with intestinal disease). Informed consent was obtained from the parents/legal caregivers prior to FOLE administration. The p-FOLE was discontinued and treatment with FOLE at 1 g/kg/day was started and infused continuously, 24 h a day, along with standard parenteral nutrition either until cholestasis resolution, full enteral nutrition was achieved, patients were transferred to another unit, or discharged to home. If higher intake of non-protein calories in parenteral nutrition was necessary, glucose intake was increased to a maximum of 18 g/kg/day.

The primary outcome was resolution of cholestasis, defined as conjugated bilirubin decreased to <2 mg/dL. Weekly measurements of conjugated bilirubin were performed until resolution of cholestasis or death. Other liver function tests were monitored as clinically considered and were measured in the clinical lab unit at HULP by spectrophotometry in an Atellica Solution platform (Siemens, Germany). In addition, from the medical records we recorded the days of life when cholestasis was diagnosed, and hemodynamic instability prior to the development of the cholestasis (septic shock, congenital heart disease on ECMO, congenital diaphragmatic hernia or ductus surgery). 

Secondary outcomes included safety aspects: death, coagulopathy (prothrombin activity <40%), and culture proven sepsis (defined as clinical findings, presence of a positive blood culture and C-reactive protein >30 mg/L). Necrotizing enterocolitis (NEC; diagnosis based on clinical signs and radiological findings—Bell’s stages 2 and 3—were considered). Intestinal perforation was distinguished from NEC based on radiographic imaging, clinical presentation, surgical findings, and medication history. Duodenal and intestinal atresia was confirmed by the surgical description. Meconium ileus was considered when the patients had issues with passing meconium and showed progressive abdominal distention and feeding intolerance despite saline enema, persistent or progressive gaseous bowel distention without high air fluid levels, or bowel wall edema noted on radiography, and patients presented with a relatively benign general condition and physiological laboratory test results. Bronchopulmonary dysplasia (BPD) was defined as needing oxygen for at least 28 days. In this study was collected moderate-to-severe BPD, being those cases with supplementary oxygen at 36 weeks postmenstrual age [8]. Intraventricular hemorrhage was assessed with sequential cranial ultrasound. 

Additionally, from the medical records, we recorded: gestational age at birth (weeks), prematurity (born <37 weeks), neonatal sex, birth weight (grams), and their percentile associated by the Fenton curves [9]. In addition, if the birth weight was below to 1000 g, the neonates were categorized as extremely low birth weight.

For the nutritional variables, we recorded: day of life when parenteral nutrition was initiated and its duration and days on Omegaven^TM^ treatment. Data on daily enteral intake were retrieved from the electronic medical records (mL/kg/day). Furthermore, the neonates with >100 mL/kg/day of enteral nutrition intake were identified. Body weight gain was defined in g/day over the NICU stay, and weight gain in grams during Omegaven^TM^ and SMOFlipid^TM^ treatment was also collected.

Data are presented for the whole cohort and comparisons were made between infants with and without resolution of cholestasis. Infants with significant gut disease, such as NEC, meconium ileus, or other intestinal diseases, were analyzed independently, as wereinfants that develop cholestasis without gut disease while receiving PN.

### 2.2. Statistical Analysis

Data analysis was performed using R software (version 4.1.1, R Core Team 2021, Vienna, Austria) within RStudio (Version 1.4.1717, RStudio, PBC, 2009–2021, Inc., Vienna, Austria) using the *dplyr*, *tidyverse*, *devtools*, *arsenal*, *compareGroups* and *rio* packages.

Data were presented as median and interquartile range (Q1; Q3) in quantitative variables or sample size (n) and relative frequency (%) in qualitative variables. The comparative between groups was performed by Mann–Whitney U test for quantitative variables and chi-squared (χ^2^) test using Fisher correction in the qualitative variables. The Spearman’s coefficient (Rho) was used to test correlation between quantitative variables. Plasma conjugated bilirubin was analyzed longitudinally, and a linear trend was created to assess the response to treatment. The significant probability was established at *p*-Value (*p*) <0.05.

## 3. Results

A total of 38 neonates received FOLE during the study period, being 44.7% (17/38) male. Gestational age at birth was 31.1 [27.0; 34.3] weeks and 92.1% (35/38) of the neonates were prematurely born, being 44.7% (17/38) extreme preterm infants (<28 weeks). Birth weight was 1390 [743.0; 2298] grams, being 47.4% (18/38) extremely low birth weight infants (Table 1). 

Regarding to clinical outcomes, prevalence of gut disease was 73.7% (28/38). Of the neonates included, 13.2% (5/38) had a hepatic disease, cholestasis was not related to any digestive system disease in 13.2% (5/38) of the cohort, and 15.8% (6/38) showed coagulopathy. Primary diagnoses of the neonates and data regarding to FOLE administration are provided in Table S1. Eleven neonates (IDs: 2, 5, 18, 25, 26, 29, 31, 33, 35, 37, and 38) did not suffer significant gut disease, although parenteral nutrition was needed.

**Figure 1:**
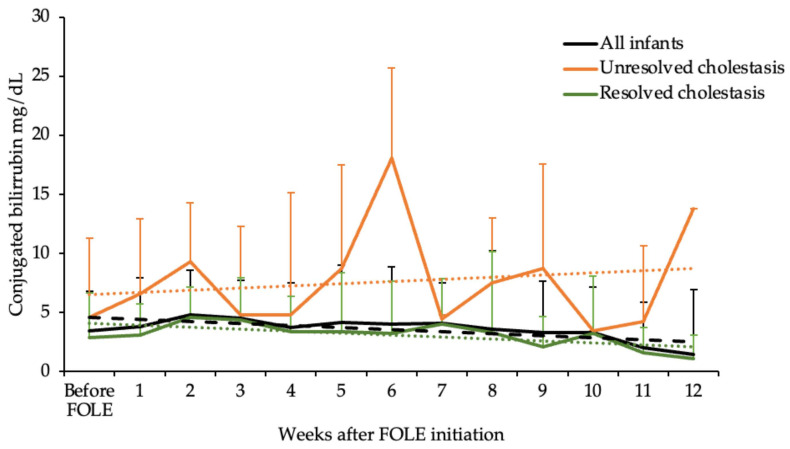
The trends of resolution for the plasma conjugated bilirubin following initiation of FOLE in all infants and separately for neonates that had cholestasis resolved versus unresolved. Data are shown as median and the positive value of the interquartile range (Q3–Q1). The discontinued line shows the decreasing of conjugated bilirubin by the linear model—black: all infants; orange: infants unresolved cholestasis; green: infants resolved cholestasis.

The age of the neonates was 15 (10; 24.8) days at cholestasis diagnosis and cholestasis resolution occurred in 73.7% (28/38) of the cohort. Plasma conjugated bilirubin levels at diagnosis was 3.8 (2.6; 6.0) mg/dL. Pattern of resolution of conjugated hyperbilirubinemia following initiation of FOLE is shown in Figure 1. In 28 infants, from 38 with cholestasis, bilirubin decreased to less than 2 mg/dL. In 34.2% (13/38) of the cohort, resolution happened before FOLE discontinuation. In these neonates, resolution happened after 29 (31.0; 69.0) days of FOLE delivery. In the rest of the cohort, the 53.6% (15/28) in which resolution occurred, they were off FOLE due to transfer to another unit (n = 5) or because there was no need of parenteral nutrition as enteral nutrition was well tolerated. These neonates received FOLE for 25.0 (8.0; 48.0) days, significantly less days of FOLE compared to those infants that resolved cholestasis before discontinuation (*p*-Value = 0.019). They were off FOLE for 27.5 (7.5; 50.5) days at the time when cholestasis resolved.

No differences in age at diagnosis of cholestasis were found between neonates with resolution (14.5 (9.8; 19.0) days) versus those who did not resolve cholestasis (23.5 (11.2; 26.5) days; *p*-Value = 0.42). Initial conjugated bilirubin levels were also similar between groups (resolve cholestasis = 3.3 (2.6; 5.7) mg/dL, unresolved cholestasis = 5.1 (3.1; 11.3) mg/dL; *p*-Value = 0.11). In addition, although both groups had the same rate of hemodynamic instability and sepsis prior to treatment with Omegaven^TM^, as expected, there was higher rate of death in the neonates who did not resolve cholestasis (80.0% vs. 3.6%; *p*-Value < 0.001, Table 1). None of them had any major bleeding as a cause of death (10.0% of the neonates died while still receiving FOLE). Death was due to sepsis in two infants( one for *Serratia marcescens*, when the infant was not receiving FOLE, and the second one for *Candida parapsilosis* after 21 days of treatment with FOLE), multiorgan failure, cardiac failure, pulmonary hypertension, or inadequacy of therapeutic effort due to bad prognosis of short bowel syndrome in very immature infants. All the infants included in this study suffered life threatening conditions with high mortality rates, but none of the causes of death were apparently related to lipid emulsion. In the infants with significant gut disease, NEC was diagnosed in the 100% of infants with unresolved cholestasis versus 50% of the infants with resolution of cholestasis (*p*-Value = 0.047).

All neonates were on parenteral nutrition with p-FOLE before starting FOLE. Parenteral nutrition was started on the first day of life (min = 0; max = 2 days). Parenteral nutrition initiation occurred earlier in infants with unresolved cholestasis (*p*-Value = 0.013, Table 2). In addition, parenteral nutrition with p-FOLE duration before switching to FOLE was 26.0 (10.0; 42.0) days. Duration of FOLE was 38.5 (11.2; 51.8) days. One of the neonates received FOLE in two courses; parenteral nutrition previously discontinued had to be resumed. Duration of parenteral nutrition was 73.5 (48.2; 106) days. Beyond prolonged parenteral nutrition, other cholestatic risk factors were frequently present in our cohort. Enteral nutrition in mL/kg/day after FOLE treatment was significantly higher in infants that showed reversal of cholestasis compared to infants in which cholestasis persisted when both groups, gut disease and non-gut disease, were analyzed independently. Furthermore, there were complications during FOLE administration in 71.1% (27/38) of the neonates such as osteopenia, hypothyroidism, adrenal insufficiency, renal insufficiency, venous thrombosis, anemia, and hyperglycemia. Sepsis was diagnosed in 81.6% (31/38) of infants after starting on FOLE (Table 2). No differences were found in sepsis incidence before and after initiation of FOLE (χ^2^ = 3.014; *p*-Value = 0.32). When number of sepsis events were corrected by days on treatment during p-FOLE, sepsis was 0.04 (0.00; 0.07) events/day, no differences was detected between infants who resolved cholestasis (0.04 (0.01; 0.08) events/day) and infants who did not resolve (0.04 (0.01; 0.04) events/day, *p*-Value = 0.36). Sepsis risk while receiving FOLE was 0.03 ((0.01; 0.04) events/day); again, no differences were detected between resolved cholestasis infants (0.03 (0.02; 0.04) events/day) and unresolved cholestasis infants (0.01 (0.00; 0.04) events/day, *p*-Value = 0.26), and no correlations were detected in sepsis events per day between *p*-FOLE and FOLE (rho = −0.03; *p*-Value = 0.84).

As expected, infants with cholestasis resolution showed lower final conjugated bilirubin than those with no resolution (Table 2). Weight gain during NICU stay was 16.0 (11.2; 21.0) g/day. No differences were found between the weight gain with FOLE and p-FOLE. 

## 4. Discussion

This retrospective and non-controlled study found that cholestasis, developed while infants received a partially fish oil-based emulsion, was resolved in a high percentage of infants after switching to a complete fish oil-based lipid emulsion (FOLE). For half of the infants in which cholestasis reversed, it occurred during FOLE administration; for the other half of the cohort, reversal occurred after FOLE discontinuation. This last group received FOLE for fewer days than those in which reversal occurred during FOLE administration; this group consisted of ten neonates when parenteral nutrition was no longer needed and five neonates although returning to p-FOLE due to be transferred to another unit. No significantly increased sepsis incidence occurred during complete FOLE administration compared with partial fish oil administration period, although the study was not powered to detect differences. 

Exclusive fish oil lipid emulsion at 1 g/kg/day has shown resolution of cholestatic liver disease in neonates, reviewed by Guthrie and Burrin [6]; compatible with our finding of resolution after discontinuation, it was found to have faster resolution once transitioned to enteral feeds. Fatty acids are critical bioactive compounds for neonatal development. Beyond nutrition and growth, lipids and their constituent fatty acids are also critical in cellular pathways that contribute to angiogenesis, organogenesis, immune response, and regulation of inflammation [10]. Structurally, they incorporate into cellular membranes and participate in regulatory activities at tissue level. Eicosapentaenoic acid (EPA) and docosahexaenoic acid (DHA) are important substrates to produce bioactive molecules including resolvins and protectins that execute their pleiotropic bioactive roles in human health and disease [10]. 

Parenteral lipid emulsions are a necessary nutritional component for newborns until adequate levels of enteral intake are established. No studies support the use of fish oils to prevent the onset of cholestasis [11,12]; this is inferred from our data, which trace the development of cholestasis in infants receiving multicomponent lipid emulsion that includes fish oil. Multicomponent lipid emulsions still contain significant phytosterol levels that correlate with plasma phytosterol concentrations [13]. Phytosterol is a cholesterol-like molecule derived from plants, similar to the cholesterol molecule but with a different side chain that may explain the positive effect of switching yo cholestasis treatment [14]. Recently, parenteral nutrition associated cholestasis has been linked with rising risks of adverse neurodevelopmental outcomes using the Bayley scale of infant development [15]. For this reason, every attempt to reverse cholestasis should be considered in the nutrition protocols of NICUs. 

An important achievement of this study is that all neonates with cholestasis and parenteral nutrition were included; we did not only consider the intestinal failure and liver disease. However, these data were provided by an observational study, which presents limitations: firstly, no causality can be proven with the current data; secondly, once intravenous lipid emulsion was not indicated by the clinician fish oil, monotherapy was discontinued; it did not remain till resolution of cholestasis; third, no control infants were analyzed. In addition, there were neonates which received FOLE for less than a week. It is apparent that conjugated bilirubin increased for one week after the initiation of FOLE, before showing a gradual decrease [7]. Results could have improved if FOLE would have been given until the resolution of cholestasis [12]. Another five infants were transferred to another unit and FOLE was discontinued before parenteral nutrition discontinuation and cholestasis was resolved. Whether this finding is due to priming with FOLE is uncertain; the natural history of cholestasis and its resolution will, in some cases, only be discerned by a randomized clinical trial comparing cholestasis resolution with FOLE versus p-FOLE.

## 5. Conclusions

In this observational study, we found that the use of fish oil monotherapy is efficacious in reversing cholestasis associated with parenteral nutrition in most neonates, independently of intestinal illness, even when it develops after receiving partial fish oil lipid emulsion and the treatment is uncompleted before cholestasis resolution. Further controlled observational and interventional studies to confirm our findings would be needed. Neonatologists should understand the composition of lipid emulsions and how they affect clinical outcomes. We now have a better understanding of FOLE, and how this emulsion reverses cholestasis.

## Figures and Tables

**Table 1:** Neonatal and clinical characteristics of the cohort.

	Total (n = 38)	Cholestasis Resolution			Infants with Gut Disease Cholestasis Resolution			Infants without Gut Disease Cholestasis Resolution		
		No (n = 10)	Yes (n = 28)	*p*	No (n = 5)	Yes (n = 22)	*p*	No (n = 5)	Yes (n = 6)	*p*
Gestational age (weeks)	31.1 [27.0; 34.3]	27.7 [25.1; 34.2]	31.6 [27.0; 34.1]	0.57	27.3 [24.4; 27.6]	31.1 [27.0; 33.7]	0.08	34.4 [33.6; 36.6]	34.4 [30.7; 35.7]	0.86
Prematurity	92.1% (35)	90.0% (9)	92.9% (26)	0.99	100% (5)	95.5% (21)	>0.99	80% (4)	83.3% (5)	>0.99
Sex (male)	44.7% (17)	40.0% (4)	46.4% (13)	0.99	20% (1)	50% (11)	0.34	60% (3)	33.3% (2)	0.57
BW (g)	1390 [743; 2298]	980.0 [707.0; 2268]	1695 [866.0; 2308]	0.45	736 [645; 830]	965 [799; 2138]	0.06	2290 [2200; 2300]	2365 [1888; 3068]	0.47
Fenton’s BW (percentile)	49.5 [22.5; 78.2]	49.5 [22.5; 65.5]	48.5 [23.2; 79.5]	0.58	51.0 [27.0; 66.0]	43.0 [10.5; 74.5]	0.85	48.0 [21.0; 64.0]	74.0 [42.8; 89.5]	0.20
ELBW	47.4% (18)	50.0% (5)	46.4% (13)	0.99	80% (4)	54.5% (12)	0.62	20% (1)	16.7% (1)	>0.99
NEC	36.8% (14)	40.0% (4)	35.7% (10)	0.99	100% (5)	45.5% (10)	0.047	0% (0)	16.7% (1)	>0.99
IVH	23.7% (9)	40.0% (4)	17.9% (5)	0.21	60% (3)	18.2% (4)	0.09	20% (1)	16.7% (1)	>0.99
BPD	50.0% (19)	70.0% (7)	42.9% (12)	0.27	80% (4)	40.9% (9)	0.17	60% (3)	33.3% (2)	0.57
Coagulopathy	15.8% (6)	30.0% (3)	10.7% (3)	0.31	20% (1)	9.1% (2)	0.47	40% (2)	16.7% (1)	0.55
Cholestasis diagnoses (DOL)	15.0 [10.0; 24.8]	23.5 [11.2; 26.5]	14.5 [9.8; 19.0]	0.42	25 [23.0; 27.0]	14.5 [10.5; 25.0]	0.15	10 [9.0; 24]	13 [8.0; 16.5]	0.78
Initial conjugated bilirubin (mg/dL)	3.8 [2.6; 6.0]	5.1 [3.1; 11.3]	3.3 [2.6; 5.7]	0.11	4.0 [2.8; 4.1]	3.1 [2.6; 4.0]	0.51	11.7 [6.0; 17.1]	4.7 [3.3; 5.9]	0.17
HA before OV^TM^	81.6% (31)	100% (10)	75.0% (21)	0.16	100% (5)	81.8% (18)	0.56	100% (5)	50% (3)	0.18
Sepsis before OV^TM^	76.3% (29)	70.0% (7)	78.6% (22)	0.67	100% (5)	81.8% (18)	0.56	40% (2)	66.7% (4)	0.57
Death	23.7% (9)	80.0% (8)	3.6% (1)	<0.001	80% (4)	5% (1)	0.002	80% (4)	0% (0)	0.015

Data show median and interquartile range (Q1; Q3) for quantitative variables and percentage and sample size (n) for qualitative variables. The *p*-Values (*p*) were extracted by chi-squared test or Mann–Whitney U test among cholestasis resolution groups. BW: birth weight; ELBW: extremely low birth weight; NEC: Necrotizing enterocolitis; IVH: Intraventricular hemorrhage; BPD: Bronchopulmonary dysplasia; DOL: days of life; HA: hemodynamic alterations; OV: Omegaven.

**Table 2:** Nutritional and ponderal body weight characteristics of the cohort.

	Total (n = 38)	Cholestasis Resolution			Infants with Gut Disease Cholestasis Resolution			Infants without Gut Disease Cholestasis Resolution		
		No (n = 10)	Yes (n = 28)	*p*	No (n = 5)	Yes (n = 22)	*p*	No (n = 5)	Yes (n = 6)	*p*
PN initiation (DOL)	0 [0; 2]	0 [0; 0]	1 [0; 5]	0.013	1 [1; 1]	1 [1; 1]	0.15	1 [1; 1]	8 [1; 13]	0.13
Days with PN	74 [48; 106]	70 [44; 112]	76 [51; 97]	0.79	64 [59; 113]	89 [70; 106]	0.71	75 [39; 108]	32 [22; 48]	0.10
Days on OV^TM^ treatment	39 [11; 52]	19 [8.0; 50]	40 [20; 55]	0.41	22 [9; 41]	46 [28; 74]	0.17	15 [14; 93]	16 [6; 23]	0.36
Sepsis with OV^TM^	81.6% (31)	60.0% (6)	89.3% (25)	0.06	100% (5)	90.9% (20)	>0.99	20% (1)	83.3% (5)	0.08
EN before OV^TM^ (mL/kg/day)	20.0 [15.0; 50.0]	16.0 [8.0; 33.0]	20.0 [15.5; 56.8]	0.40	0.0 [0.0; 16]	0.0 [0.0; 11.8]	0.84	0.0 [0.0; 0.0]	7.5 [0.0; 39.0]	0.08
EN after OV^TM^ (mL/kg/day)	90.0 [30.0; 110]	0.0 [0.0; 0.0]	92.5 [60.0; 110]	0.10	0.0 [0.0; 0.0]	77.5 [20.8; 100]	0.002	0.0 [0.0; 0.0]	105 [77.5; 110]	0.004
^1^ EN >100 mL/kg/day	61.1% (22)	40.0% (4)	69.2% (18)	0.14	50% (2)	65% (13)	0.62	40% (2)	83.3% (5)	0.24
DOL when EN >100 mL/kg/day was reached	47 [30; 61]	43 [28; 57]	47 [30; 85]	0.73	27 [25; 28]	50 [40; 92]	0.09	57 [56.5; 57.5]	30 [19; 30]	0.05
Finish OV^TM^ (DOL)	64 [44; 100]	60 [46; 101]	67 [46; 96.0]	0.80	63 [57; 93]	71 [61; 107]	0.55	56 [43; 133]	35 [22; 46]	0.10
Final conjugated bilirubin (mg/dL)	1.3 [0.7; 1.98]	4.5 [4.1; 11.1]	1.1 [0.58; 1.6]	<0.001	4.2 [3.67; 7.92]	0.97 [0.52; 1.30]	0.002	10.4 [4.5; 11.1]	1.58 [1.29; 1.67]	0.006
BWG (g/day)	16.0 [11.2; 21.0]	14.8 [12.2; 21.0]	16.0 [10.3; 21.0]	0.80	14.5 [12.0; 15.0]	16.0 [11.8; 21]	0.20	22.0 [13.0; 28.0]	16.4 [9.6; 16.9]	0.41
WG with OV^TM^ (g)	70.2 [20.5; 154]	31.0 [10.8; 133]	79.2 [27.2; 155]	0.34	22.0 [8.8; 66.3]	105 [35.2; 165]	0.11	40.0 [17.1; 173]	42.2 [9.8; 79.2]	0.47
WG with SMOFlipid^TM^ (g)	67.0 [30.2; 139]	56.9 [34.5; 102]	71.5 [28.2; 156]	0.95	68.0 [41.8; 114]	79.5 [44.5; 177]	0.90	47.8 [32.0; 66.0]	28.5 [9.3; 94.5]	0.47

Data show median and interquartile range [Q1; Q3] for quantitative variables and percentage and sample size (n) for qualitative variables. *p*-Values (*p*) were extracted by chi-squared test or Mann–Whitney U test among cholestasis resolution groups. PN: Parenteral nutrition; DOL: days of life; BWG: body weight gain; WG: weight gain; EN: enteral nutrition; OV: Omegaven, ^1^ from start of PN to the end of OV^TM^.

## Data Availability

The data presented in this study are available on request from the corresponding author. The availability of the data is restricted to investigators based in academic institutions.

## Supplementary Materials

The following supporting information can be downloaded at: https://www.mdpi.com/article/10.3390/nu15030509/s1, Table S1: Neonate primary diagnoses, FOLE administration and outcomes.
