# Supplementary material for: Exclusive Fish Oil Lipid Emulsion Rescue Strategy Improves Cholestasis in Neonates on Partially Fish Oil-Based Lipid Emulsion: A Pilot Study
Source: Nutrients. 2023 Jan 18;15(3):509. doi: 10.3390/nu15030509 (PMC9920606; doi:10.3390/nu15030509)
Supplement: Supplementary file 1 [file nutrients-15-00509-s001.zip › nutrients-2051737-supplementary.pdf]

Table S1. Neonate primary diagnoses, FOLE administration and outcomes.

ID	Gestational age (weeks+days)	Birth weight (grams)	Primary diagnosis	FOLE initiated (DOL)	Days on FOLE	Cholestasis resolution	Cholestasis resolution (days after FOLE initiation)
1	23+6	702	Meconium ileus<br>NEC	38	121	Yes	120
2	24+1	697	Fungal sepsis	42	6	No	-
3	24+3	645	NEC	29	11	No	-
4	24+3	736	NEC	41	22	No	-
5	25+2	487	Hepatic hematoma	10	40	Yes	40
6	25+2	686	Intestinal obstruction	30	11	Yes	14
7	25+6	939	NEC	54	8	Yes	20
8	26+0	910	Intestinal atresia	94	12	Yes	71
9	26+6	731	Intestinal obstruction	25	47	Yes	48
10	27+0	905	NEC	26	79	Yes	42
11	27+0	644	Meconium ileus	21	4	yes	18
12	27+2	1130	NEC	48	73	No	-
13	27+3	900	NEC	61	48	Yes	50
14	27+4	919	Intestinal perforation	26	90	Yes	120
15	27+4	830	NEC	52	49	No	-
16	27+6	455	Sepsis	53	5	No	-
17	28+0	991	NEC	51	52	Yes	29
18	29+5	1740	Polymalformative syndrome	14	6	Yes	64
19	30.6	1820	NEC	8	75	Yes	78
20	31+2	1952	NEC	25	69	Yes	64
21	31+6	1650	NEC	57	40	Yes	31
22	32+0	2300	Volvulus	36	31	Yes	32
23	32+0	765	Meconium ileus	10	37	Yes	41
24	33+2	2374	Intestinal atresia	6	63	Yes	34
25	33+4	2300	Neck-chest mesenchymal tumor	28	15	No	-
26	33+5	2400	Hepatic hemangioendothelioma	21	27	Yes	70
27	33+6	650	Intestinal perforation	41	46	Yes	11
28	33+6	2500	Gastroschisis with intestinal atresia	19	95	Yes	23
29	34+3	2290	Congenital Diaphragmatic Hernia	20	7	No	-
30	35+0	1750	Gastroschisis; intestinal atresia; volvu-<br>lus	20	51	Yes	180

31	35+1	3410	Hepatoblastoma	7	23	Yes	98
32	35+4	2245	Duodenal atresia	17	47	Yes	56
33	36+4	2200	Omphalopagus twin (biliary atresia)	7	96	No	-
34	36+4	2200	Omphalopagus twin (Intestinal atresia and perforation)	12	23	Yes	21
35	3+6	2330	Viral hepatitis	13	4	Yes	46
36	37+1	2470	Hirschsprung disease	9	10	Yes	9
37	38+4	3290	Sepsis	26	24	Yes	23
38	39+4	3025	Shone complex	40	50	No	-

DOL = day of life; FOLE = 100% fish oil–based lipid emulsion; NEC = necrotizing enterocolitis
